# The 19th St. Gallen international breast cancer conference ‘primary therapy of patients with early breast cancer. Evidence, controversies, consensus’: key moments and breakthroughs

**DOI:** 10.3332/ecancer.2026.2075

**Published:** 2026-02-13

**Authors:** Consuelo Morigi

**Affiliations:** Division of Breast Surgery, IRCCS European Institute of Oncology, 20141 Milan, Italy

**Keywords:** 19th St Gallen consensus conference 2025, SGBCC 2025, early breast cancer, neoadjuvant chemotherapy, adjuvant therapies, surgery, trials, artificial intelligence, consensus

## Abstract

The 19th St. Gallen International Breast Cancer Conference took place at the Austria Centre in Vienna from March 12th to 15th, 2025. The event achieved remarkable success, drawing over 3,100 participants from all over the world. This accomplishment is attributable to the high calibre of scientific lectures presented by leading global experts in breast cancer (BC) treatment, the opportunity for the audience to interact with panellists through questions, the interesting and varied scope of submitted posters, the debates that punctuated the many sessions, and notably, the renowned St. Gallen Consensus Session. This year's Opening Ceremony held particular significance. In commemoration of the Swiss physician and oncologist Hansjoerg Senn, who passed away in 2023, the Hansjoerg Senn Memorial Lecture was instituted to honour his profound contributions to the treatment and care of patients, particularly those affected by BC. The Memorial Lecture serves as a platform to recognise and celebrate individuals who have significantly advanced BC research. The recipient is presented with the St. Gallen Breast Cancer Award and invited to deliver an address at the opening ceremony of the St. Gallen International Breast Cancer Conference. This year, this prestigious award was presented to Prof. Armando E. Giuliano, who delivered an insightful lecture entitled ‘My Personal Experience: Changing Surgery for BC.’ In it, drawing from his impressive surgical career during which he changed the standard of care—exemplified by the sentinel lymph node procedure and the Z0011 study—he spoke about how change, though difficult, is both necessary and inevitable, emphasising that no one is too young or too old to change.

## Ouverture: glancing into the future—moving up innovative agents from metastatic breast cancer (BC) to early BC

*G. Curigliano* began his presentation by emphasising that the primary goal in the early-stage BC setting is to improve overall survival (OS). Can we improve OS? Looking at the data, the answer is yes. This is due to improved diagnosis and enhanced local and systemic treatment [1]. Regarding systemic treatment, it is interesting to note that for some drugs, such as pembrolizumab, the timeframe between approval in the metastatic setting and the early setting was very short (3 years), indicating that data from the metastatic setting can inform trial design in the early setting, potentially translating into improved OS. Validated surrogate endpoints are essential to accelerate drug development and regulatory approval. A surrogate endpoint should be associated with the true endpoint at both the patient level (regardless of the treatment arm) and the trial level (considering the real treatment effect). The association between these two is measured by the coefficient of determination *R*^2^. To establish a good correlation between the surrogate endpoint and OS, *R*^2^ must be ≥0.7 [2, 3]. While awaiting OS data, the mechanism of action of novel agents and the selection of appropriate intermediate endpoints can help inform patient outcomes and guide treatment decisions in clinical practice.

F. Andre spoke about novel endocrine agents. There are numerous ongoing trials on the use of adjuvant oral selective estrogen receptor degraders (SERDs) in early BC that will help clarify various aspects (e.g., CAMBRIA-1, EMBER-4 and lidERA). To ensure the proper use of SERDs, within the framework of personalised patient therapy, it will be crucial to define the risk of relapse, utilising new tools such as artificial intelligence (AI) and circulating tumour DNA (ctDNA). Moreover, it will be important to also consider patient preferences and their quality of life (QoL). It is well known that endocrine therapy (ET) is associated with a clinically relevant decrease in QoL [4]. We need to determine if SERDs improve QoL compared to aromatase inhibitors (AIs). In BC, it should be noted that non-adherence to adjuvant ET constitutes a major obstacle to optimal outcomes; the primary cause of this non-adherence is toxicity [5]. Regarding ESR1 mutations (ESR1m), they are rarely acquired during adjuvant therapy with AIs, but frequently during AIs therapy in the metastatic setting [6]. In the latter case, SERDs are effective in patients with ESR1m [7] and have similar efficacy compared to fulvestrant in patients with ESR1 wild type after the onset of resistance to ET in the metastatic setting [8]. It will be necessary to understand if SERDs are capable of preventing resistance mechanisms not related to ESR1m. SERDs also appear to be effective in ET-naive patients with early BC, with a statistically significant reduction in KI-67 when compared to that caused by AIs [9]. It will be interesting to analyse whether SERDs, like AIs, have a ‘prognostic’ value based on the response to preoperative ET [10].

J Cortes focused on new anti-HER2 approaches. The largest improvements in HER2+ BC have been achieved in the metastatic setting, thanks not only to the introduction of Trastuzumab [11] but also to several studies demonstrating progression-free survival (PFS) benefits: CLEOPATRA with the addition of pertuzumab [12], EMILIA with trastuzumab emtansine (T-DM1) compared to lapatinib plus capecitabine [13], HER2CLIMB with the addition of tucatinib to trastuzumab and capecitabine [14] and Destiny-Breast 03 with trastuzumab deruxtecan (T-DXd) compared to T-DM1 [15]. An important aspect to consider when moving from trials in the metastatic setting to early BC is patient selection based on clinical risk. Furthermore, patient selection based on response to therapy, such as pathological complete response (pCR), is crucial for tailoring therapies (e.g., COMPASS trial and Destiny-Breast 05). Randomised clinical trials have contributed enormously to advances in patient care. Nevertheless, they have several shortcomings, including the need for a large sample size and long study duration, the lack of power to evaluate efficacy overall or in important subgroups and cost. To address these limitations, adaptive trial designs appear to be the best way to move forward towards de-escalation treatment-based strategies.

S. Tolaney discussed antibody-drug conjugates (ADCs): an innovative family of agents assembled through linking cytotoxic drugs (payloads) covalently to monoclonal antibodies to be delivered to tumour tissue that expresses their particular antigen, with the theoretical advantage of an augmented therapeutic index. To date, ADCs approved in HER2+ metastatic BC are: Sacituzumab govitecan, Datopotamab deruxtecan, T-DXd and Sacituzumab tirumotecan. None of these drugs is currently approved for early BC, but with the intention of expanding their indications, several ongoing trials are underway, such as the NeoSTAR trial, TROPION-Breast04 and I-SPY 2 for use in the neoadjuvant setting, TROPION-Breast03 and SASCIA (NCT04595565) trials for patients who do not achieve pCR after neoadjuvant chemotherapy (NACT) and ASPRIA (NCT04434040) and SURVIVE HERoes (NCT06643585) trials, which rely on the presence of ctDNA post-adjuvant therapy to select patients for ADC use. In the future, it will be necessary to better understand several aspects, including whether ADCs can be sufficient to replace chemotherapy (CT), whether it is better to administer them in sequential or combination regimens, and to analyse their toxicity.

## Session I: Liquid biopsy

N. Turner presented the technology for detecting ctDNA and its important role in trials and clinical practice, emphasising the necessity of using appropriate assays. Assay types for early-stage cancer ctDNA are essentially of two kinds: tumour-informed assays, which require tumour tissue to track patient-specific mutations, and tumour-agnostic assays, which target predefined signatures allowing broader applicability but with relatively lower sensitivity. Regarding tumour-informed assays, they can offer high precision in minimal residual disease (MRD) detection. Studies suggest that detection of ctDNA during follow-up is associated with a high risk of future relapse of early-stage BC, with a good temporal window over clinical relapse [16]. Regarding agnostic assays, the test panel is the same for all patients; thus, even using an advanced BC genotyping assay, if ctDNA is detected, it certainly has prognostic value but with lower discriminatory potential than tumour-informed assays. As a result, sensitivity will be lower than with tumour-informed tests [17]. Conversely, ctDNA detection with genotyping assays or circulating tumour cell (CTC) analysis shows a lack of specificity. Regarding CTCs, finding high levels of CTCs is prognostic, but high levels of CTCs are rare; low levels are more common and have limited prognostic significance [18]. Genotyping assays are not specific for the presence of clonal haematopoiesis of indeterminate potential (CHIP), which is the acquisition of somatic mutations leading to clonal expansion of hematopoietic stem cells and is a common incidental finding in ctDNA, especially in older individuals. This has the potential to seriously undermine tumour-agnostic ctDNA detection; in fact, in large sequencing panels, >50% of mutations are from CHIP instead of cancer [19]. The clinical significance of CHIP warrants further study.

F.

C. Bidard analysed the role of ctDNA in screening/diagnosis of early BC. In this setting, the difficulties include: robust performance of imaging, ease of diagnostic biopsies and the fact that non-invasive and small lesions shed very few circulating biomarkers. PATHFINDER is a pilot study investigating the feasibility of Multicancer Early Detection (MCED) blood tests, which can identify a cancer signal from circulating cell-free DNA. His experience lays the foundation for larger-scale studies to evaluate the clinical effectiveness of MCED testing as a cancer screening strategy [20]. ctDNA-based detection of MRD presents a strategy to identify patients at high risk of relapse. First-generation tests were only marginally prognostic; conversely, the whole-genome sequencing-based assay resulted in improved ctDNA detection at diagnosis and long lead times (median lead time 15 months) between MRD detection and relapse. These findings merit additional investigation through further prospective studies that can establish the clinical impact of this method throughout the diagnostic and therapeutic pathway [21]. There are several ongoing trials to explore the clinical utility of ctDNA (e.g., CUPCAKE, PERSEVERE, KAN-HER2, TREAT-ctDNA, DARE, TRAK-ER and SURVIVE-HERoes trial).

W. Janni continued to describe the topic of including ctDNA in clinical trials. In advanced BC, for example, the use of ctDNA can represent a predictive biomarker to guide treatment. The Serena 6 trial aims to detect ESR1m in ctDNA. ESR1m is a frequent cause of acquired resistance to AIs plus cyclin-dependent kinase 4 and 6 inhibitors (CDK4/6i). The purpose of this trial is to evaluate the efficacy and safety of switching from an AIs to camizestrant (SERD), while maintaining the same CDK4/6i, upon detection of ESR1m in ctDNA before clinical disease progression. The primary endpoint in this case is PFS. In early BC, surrogate markers of OS are represented by invasive disease-free survival (iDFS) and, after NACT, pCR. However, even if the achievement of a pCR after NACT identifies patients with a low risk of recurrence, some of them still relapse. Conversely, not all patients with residual tumour after NACT have a recurrence of disease. This highlights the need for additional biomarkers for a more accurate stratification of the risk of recurrence, and the role of ctDNA appears promising [22].

## Session II: HER2+ early BC—mission accomplished?

M. Piccart reflected on the role of trastuzumab on the 20th anniversary of its clinical use, noting that after all these years, it still remains the ‘queen’ in terms of long-term efficacy, safety and global accessibility. Many important studies have been conducted on this monoclonal antibody, which have shown that 1 year of adjuvant trastuzumab for HER2+ BC significantly improves long-term disease-free survival (DFS) (HERA trial) [11, 23], that concomitant CT + trastuzumab is better than a sequential regimen (N9831 trial) [24-25] and that adjuvant trastuzumab with a non-anthracycline-based regimen is very effective and also less cardiotoxic (BCIRG006 trial) [26]. Several studies have been conducted to evaluate different durations of trastuzumab. For example, the HERA trial clarified that 2 years had no additional benefit, the PERSEPHONE trial demonstrated that 6 months of trastuzumab treatment is non-inferior to 12 months of treatment in patients with HER2+ early BC, with less cardiotoxicity and fewer severe adverse events [27]. Conversely, the PHARE trial did not show the non-inferiority of 6 months versus 12 months of adjuvant trastuzumab [28], nor the HELLENIC trial, which showed a higher DFS in favour of the 12-month standard treatment [29]: at present, the standard of care is still 12 months of treatment. In the near future, we will likely benefit from the use of the HER2DX test to personalise the treatment of patients with HER2-positive BC.

C. Denkert focused her presentation on HER2 testing after 20 years. American Society of Clinical Oncology (ASCO)/College of American Pathologists (CAP) established consistent guidelines for HER2 testing, which are essentially conservative. In the 2023 update, HER2-low and HER2-ultralow were not included as interpretive categories. Over the years, the concordance of HER2 testing among pathologists has increased, thanks to, for example, the use of two independent methods, ASCO/CAP guidelines and standardised EQA programs. This concordance has now reached more than 90%, but there is room for further improvement in the HER2-low category. Furthermore, the discordance rate is higher in ER+ BC than in the ER-subgroup. This could be explained by the RNA results, which show a bimodal distribution of HER2 mRNA in ER- BC and a continuous distribution in ER+ BC. Within the continuous distribution, it is much more difficult to determine a cutpoint [30]. This is consistent with the fact that within the HER2-low category, 60% of patients are ER+. What is the minimum level of HER2 that is necessary for Trastuzumab response? IHC 3+ or FISH amplified [31]. Regarding T-DXd response, in the metastatic setting, it has shown consistent clinical benefit not only in IHC 3+ but also in HER2-low [32] and HER2-ultralow [33]. PIK3CA mutations can be found in about 20%–30% of HER2+ BC and indicate a lower response to CT and anti-HER2 therapy, especially in ER+/HER2+ BC. The GeparPiPPa trial investigates, in the neoadjuvant setting, the clinical utility of inavolisib, an oral PI3Kα inhibitor, in addition to ET and trastuzumab/pertuzumab in early ER+/HER2+ and PIK3CA mutant BC. Recently, data on molecular subtypes of HER2 with prognostic value have been published [34]. All this information will help to better tailor therapies for HER2+ BC in the near future. N. Harbeck's presentation discussed risk-adapted neoadjuvant and adjuvant therapy for HER2+ BC. Specifically, for T1, N0 tumours, upfront surgery is reliable. After surgery, 12 weeks of paclitaxel + trastuzumab followed by 40 weeks to complete a full 1 year of trastuzumab is a reasonable treatment (APT trial) [35]. For tumours > pT1, pN0, adjuvant polychemotherapy plus 1 year of trastuzumab in N0 or dual HER2-blockade (trastuzumab + pertuzumab) in N+ is indicated. In fact, with an 8-year follow-up, the N+ subgroup maintained a significant iDFS benefit favouring dual blockade, without significantly improving OS; no benefit was seen in the N0 subgroup (APHINITY trial) [36, 37]. For tumours > 2 cm or N+, NACT with trastuzumab and pertuzumab is recommended. In the case of pCR, which has prognostic value [38], omission of further CT did not affect iDFS; these patients should continue anti-HER2 therapy for a total duration of 1 year [39]. There are de-escalation trials based on early response, such as PHERGAIN, which is a PET-based, pCR-adapted strategy capable of identifying patients with HER2+ BC who could safely omit CT [40] or TRAIN-3, in which a radiological complete response evaluated via magnetic resonance imaging (MRI) can limit the duration of NACT [41].

K. Jhaveri discussed how to manage residual disease after NACT in HER2+ BC. The current standard of care for patients with HER2+ BC and no-pCR after NACT is replacing trastuzumab with T-DM1 for 14 cycles (KATHERINE trial), which, after a median follow-up of 8.4 years, demonstrated a significant benefit in iDFS and OS [42, 43]. Data from the DESTINY-Breast05 trial in early HER2+ BC are awaited to understand if it is possible to replace T-DM1 with T-DXd, which has shown benefits in the metastatic setting. Another option that might offer a potential opportunity in patients with HER2+ BC who did not achieve pCR after NACT is to add to trastuzumab another agent, such as tyrosine kinase inhibitors like Neratinib for 1 year (ExteNET trial), which showed a significant increase in iDFS and OS in ER+/HER2+ patients [44]. However, we do not have data in patients who have previously received pertuzumab and/or T-DM1, and it is necessary to evaluate the benefit-to-toxicity ratio, as Neratinib is associated with high rates of moderate/severe diarrhoea [46]. In the COMPASSHER2 trial, the addition of Tucatinib to T-DM1 is being tested, and in the ASTEFANIA trial, the addition of Atezolizumab to T-DM1. The binary division between pCR and non-pCR is insufficient; in fact, it is more significant to consider the residual cancer burden (RCB), which has been shown to correlate better with prognosis [45].

## Session III: Minimising the burden of cancer treatments: more tailoring for early BC

**C**. Coles spoke about the potential safety of omitting radiotherapy (RT) or ET. For patients with low-risk, early-stage BC, offering standard adjuvant treatment with RT + ET ensures excellent oncological outcomes, but it may constitute overtreatment. Scientific data indicate that ER or ET are likely viable single-modality treatments and that RT has a more favourable tolerability profile, but definitive conclusions will depend on long-term disease control outcomes [47, 48, 49]. Regarding surgical treatment, studies are also investigating the feasibility of avoiding surgery, using instead minimally invasive treatments, such as vacuum-assisted excision [50], radiofrequency ablation [51] or cryoablation [52]. Validated biomarkers can help identify patients at very low risk of recurrence and better plan a potential de-escalation of treatments.

H. Wildiers clarified the use of CT in older patients. It is certain that elderly patients do not require the same treatments as the younger population for various reasons, such as lower life expectancy, greater potential for toxicity and more pre-existing comorbidities. However, it is also true that there is huge heterogeneity in health status among these patients. There are tools to predict severe toxicity events that can aid in the decision process with older patients, such as ARG-BC, AGE-GAP and PORTRET. The data in the literature tell us that CT should not be denied to older BC patients solely because of their advanced age, but in general, only very fit older patients can be treated with sequential regimens used in younger patients. Instead, docetaxel + cyclophosphamide (TC) is a feasible regimen for the majority of older patients [53, 54]. For HER2+ early BC, Trastuzumab can be combined with the TC regimen, but Granulocyte colony-stimulating factor (G-CSF) is recommended to avoid febrile neutropenia. In frail patients, weekly paclitaxel can be considered as a regimen to combine with trastuzumab. For unfit selected patients, as highlighted by the RESPECT trial, BC adjuvant trastuzumab monotherapy can be considered [55]. In case of toxicity, it is better to evaluate a shorter duration of trastuzumab [56]. Data did not find a statistically significant increase in OS with the addition of CT to ET after surgery for high-risk ER+/HER2-BC [57]. The results of the APPALACHES trial will clarify whether these patients can benefit from the addition of CDK4/6i (Palbociclib) to ET as an alternative to CT. In the search for a balance between overtreatment versus undertreatment, in a way, every oncologist should become a geriatric oncologist, evaluating a personalised treatment for each individual patient, also considering the patient's preferences.

M. Oliveira underlined the need to understand the right balance between the pros and cons of the many new approaches we have available for BC care. For example, starting from the consideration that clinical trials often involve a selected patient population in a controlled environment, which may clash with the real-world environment, sometimes characterised by limited resources and patient populations not included in clinical trials. Moreover, beyond the efficacy endpoints of a treatment, it is necessary to consider the patients' QoL, potential toxicity, drug costs and, in general, the global burden on the healthcare system. An aid in these evaluations can come from the use of, for example, the European Society for Medical Oncology (ESMO) Magnitude of Clinical Benefit Scale. An important aspect in the era of precision medicine is certainly the introduction of biomarkers, which can allow better patient stratification in clinical trials, identification of response and toxicity to new drugs and definition of predictive recurrence risks on which to tailor therapeutic strategies.

## Session IV: Optimising locoregional treatments of the breasts

Why should we prevent mastectomies? J. De Boniface, in his presentation, provided a compelling answer to this question. Mastectomy should be avoided when breast-conserving surgery (BCS) is a viable option. This is due to several critical factors. First, mastectomy does not consistently guarantee oncologic safety. In fact, 94% of mastectomy specimens, particularly in so-called conservative mastectomies, may contain residual glandular tissue within the skin flaps. Consequently, this procedure should be undertaken by surgeons with a higher level of expertise. Second, there is a lack of survival benefit; there are indeed no randomised controlled trial data that demonstrate a significantly improved survival advantage with mastectomy compared to BCS. We should additionally consider that mastectomy is associated with a potentially more elevated risk of complications, and some studies suggest that these complications may correlate with poorer survival outcomes [58]. Mastectomy does not always result in improved patient-reported outcomes regarding satisfaction with breasts, even when combined with reconstruction [59, 60]. We must also consider that mastectomy is often related to reconstructive challenges and furthermore, it often necessitates additional symmetrisation procedures, resulting in further scarring. Therefore, the answer to the initial question appears clear: we must strive to prevent unnecessary mastectomies and offer oncoplastic BCS as an alternative to mastectomy in all women in whom it is technically feasible, while also actively involving the patient in the decision-making process.

D. Kauer-Dorner discussed tailoring RT. Historically, whole-breast irradiation with traditional fractionation, consisted of 50 Gy in 25 fractions over 5 weeks, it was the standard of care for several years. Partial breast irradiation (PBI), as compared with WBI, is associated with less acute toxicity but with no significant difference in late toxicity [61]. PBI is an evidence-based treatment option to reduce the burden of RT in selected low-risk patients aged 50 and above, with unifocal, unicentric tumours <3 cm, pN0 and resection margins of at least 2 mm [62].

Moderate hypofractionation (15 × 2.67 Gy) in 3 weeks should be the standard treatment for all indications of modern postoperative RT in BC. In fact, results from many trials (e.g., START-B, FABREC, DBCG HYPO, IMPORT-HIGH and HypoG-01) have shown no differential effect of fractionation schedule for tumour control by age, type of primary surgery, eventual reconstruction, axillary node status, use of adjuvant CT or association of boost RT. Ultra-hypofractionation (5 × 5.2 Gy) in 1 week is becoming a new opportunity, especially for elderly patients (FAST-FORWARD). An extension of indications will likely come from the results of ongoing trials (e.g., FAST-FORWARD nodal and Hyport-Adjuvant). RT is important for local control even in ductal carcinoma *in situ* (DCIS), but, in this case, probably the current challenge is to identify the risk of recurrence in individual patients to potentially omit RT. In this context, prospective validation of novel biomarker assays in DCIS is needed (Oncotype DX DCIS Score and DCISionRT Decision Score). The personalisation of local therapy is obviously important also in invasive BC, and there are many trials that, using gene signatures (e.g., Oncotype DX and Prosigna PAM50), are evaluating the omission or addition of RT (e.g., IDEA and PRECISION). Therefore, omitting RT in low-risk patients should be part of a shared decision-making process with our patients.

V. Galimberti presented on breast surgery after NACT. The goals of NACT for surgery are: de-escalation of breast surgery, by increasing BCS and reducing the rate of axillary lymph node dissection (ALND). Regarding margins, the optimum is to remove all known residual rather than the original tumour lesion with a ‘no ink on tumour’ resection, regardless of the presence of unifocal or multifocal disease [63, 64, 65]. Residual microcalcifications, however, may present a clinical and surgical challenge. Complete excision of tumour bed calcifications remains standard practice and a potential limitation to NACT use for downstaging patients to be suitable for BCS. In fact, although calcifications seen on post-NACT mammography might not be associated with persistent disease, the loss of MRI enhancement and changes in calcifications do not predict the absence of residual tumour with sufficient accuracy [66, 67]. In the near future, it is desirable to be able to avoid surgery in patients with pCR after NACT. What are the safest criteria for selecting patients in clinical trials to avoid breast surgery after NACT? The rate of pCR is higher in triple-negative breast cancer (TNBC) and HER2+ disease, and pCR after NACT is associated with significantly better DFS and OS [68]. Conventional imaging lacks sufficient sensitivity/specificity to predict pCR, while MRI-radiomics has emerged as a potential method for predicting the response to NACT in BC patients, showing promising outcomes [69]. The use of image-guided vacuum-assisted biopsy to obtain at least six representative samples of residual breast imaging allows reliable prediction of residual disease [70]. There are many ongoing trials on the omission of surgery after NACT (e.g., BETTY-CRASY, OPTIMISTC, JCOG1806:AMATERAS-BC and ELPIS), but in the meantime, the first results obtained in the EXCEPTIONAL RESPONDERS trial highlight that, in highly selected patients, omission of surgery after NACT was associated with an ipsilateral breast tumour recurrence-free survival of 100% [71].

C. Solbach discussed breast reconstruction, which should be an individualised decision for each patient, seeking to understand the expectations for breast reconstruction and in agreement with the healthcare team, also considering the treatments that the patient will have to undergo, in particular RT. Comprehensive information and support throughout the decision-making process is essential to ensure that patients make informed decisions that reflect their personal values and expectations. AI can further aid in this collaborative decision between physician and patient (CINDERELLA trial).

## Session V: Nuances in adjuvant therapy for ER+ BC

D. Cameron gave an overview of the standard and new drugs we have available for the treatment of ER+ BC BC. Today, we have many drugs available, such as Tamoxifen (TAM), AIs, luteinizing hormone-releasing hormone analogues and CDK4/6 inhibitors. Regarding these latter drugs, which were introduced more recently, when offering this treatment to patients, it is necessary to inform them that, at the moment, this treatment offers a minimal residual risk of relapse (16%), in exchange for frequent side effects such as fatigue, diarrhoea and worse alopecia. Sadly, endocrine studies take time to mature OS data. However, we need to make sure we are delivering better long-term outcomes.

Some ER+ BC still require CT, as discussed by K. Kalinski. First, it is necessary to consider it if the benefit provided by CT is at least 5% The TAILORx trial showed similar efficacy of adjuvant ET versus CT plus ET in patients with early-stage BC and a 21-gene recurrence score (RS) of 11 to 25. However, there is a subgroup of patients younger than 51 years, with RS 16–25, and high clinical risk, who derived some CT benefits [72]. Conversely, in patients with a high RS of 26 to 100, the addition of CT improves the rate of freedom from recurrence [73]. Clinical studies have shown that patients with RS ≥ 31 had better outcomes when treated with adjuvant anthracycline plus taxane-based CT regimens compared with those receiving adjuvant taxane-based CT regimens alone. A subgroup analysis showed benefit even for tumours larger than 2 cm, regardless of menopausal status [74]. Among premenopausal women with one to three positive lymph nodes and an RS of 25 or lower, those who received chemoendocrine therapy had longer iDFS and DRFS than those who received ET only, whereas postmenopausal women with similar characteristics did not benefit from adjuvant CT [75]. An analysis of the RxPONDER trial found that premenopausal women with BC who had low levels of anti-Müllerian hormone (AMH) benefited less from CT adjunctive to ET than women with medium or high levels of AMH. AMH appears to be a stronger predictor of CT response than self-reported menopause status and age [76]. The MINDACT trial showed that in BC patients with high clinical and low genomic risk, the benefit of adding CT compared to And *et al*one is low, except in women younger than 50 years, defining a CT benefit that differs by age [77]. Most premenopausal patients in the TAILORx and RxPONDER trials did not receive ovarian function suppression (OFS) as part of their ET regimen. Given the observed benefit from OFS in high-risk premenopausal patients with homologous recombination (HR)+/HER2-BC in the SOFT/TEXT trials, many questioned whether all or part of the observed CT benefit in the TAILORx/RxPONDER trials may have been the result of CT-induced OFS. The ongoing trial BR009 will attempt to answer this. There are other tools, such as RSClinN+, which seem more prognostic than RS or clinicals alone, to individualise recurrence risk and CT benefit predictions [78].

P. Francis considered individualised ET in premenopausal women. In these patients, there are several factors to consider, such as age (especially very young patients), tumour stage and biology, multigene assay results, other planned systemic treatments and even interruption of treatments for desire of pregnancy (POSITIVE). For example, if in low-risk patients we can consider offering TAM for 5 years (EBTCG), as the risk of recurrence increases, we will need to modulate the treatment, for example, by increasing the duration of TAM (ATLAS), switching to aromatase inhibitors AIs (MA.17) or adding OFS (ASTRRA, SOFT and TEXT) [79]. Furthermore, there are several other drugs concurrent with ET to reduce the risk of recurrence, such as the addition of Abemaciclib for 2 years to any ET (monarchE) [80], Letrozole (or Anastrozole) + OFS + Ribociclib for 3 years (NATALEE), Any ET + Olaparib for 1 year (OLYMPIA) and OFS + Zolendronate (ABCSG 12).

T. Mamounas went into more detail regarding the duration of ET. We know that clinical and pathological factors of BC are prognostic [81], and therefore, the duration of ET should take these aspects into consideration. In the literature, there is a lot of data in favour of extending TAM or AIs (e.g., ATLAS, MA.17, MA17R, B-42 and DATA). However, ET can often have toxic effects and impact the patients' QoL. The ideal scenario would therefore be to predict the benefit of extended endocrine therapy (EET). In this, the use of clinic-pathological algorithms such as CTS5 [82], the monitoring of ctDNA after surgery, which has emerged as an accurate predictor of BC recurrence [83], and genomic assays such as breast cancer index [84, 85] or Mammaprint [86] can help. These factors will become increasingly important in helping to decide on potential EET, especially in this era of axillary surgical de-escalation.

And what about invasive lobular carcinoma (ILC)? M.J. Vrancken-Peeters discussed this topic. Routine use of MRI seems to improve the detection of multifocality and bilateral BC, even though it can lead to higher rates of false-positive findings and overestimation of tumour size [87]. However, in upfront surgery, it has shown a significant six-fold reduction in reoperations [88]. Residual lobular tumour size after NACT is often underestimated by MRI [89, 90]. Regarding staging, ILC demonstrates lower visibility on FluoroDeoxyGlucose-positron emission tomography than the more common invasive ductal carcinoma (IDC), but promising data seem to exist for the alternative use of Fluoroestradiol F18-Positron emission tomography; larger trials are needed for better evaluation [91]. Regarding de-escalation of axillary surgery, it is probably feasible, but further data are needed, as we know that ultrasound (US) has limitations in detecting lymph node metastases in this subgroup of patients, and in the major trials (SOUND, INSEMA), the percentage of patients with ILC BC is reduced. Patients with ILC who have a high RS are treated less often with CT compared with similar patients who have IDC, but the role of RS is relevant in these patients [92].

S. Zhiming considered the importance of targeted therapy in ER+ BC. Luminal BC presents a wide heterogeneity, and in this case, the basis for precision treatment, such as EET and evaluation of traditional or emerging treatments could be represented by the integration of clinical, transcriptomic, metabolomic, radiomic and pathological features. This allows for efficient stratification of patients into groups with different recurrence risks and different subtype-specific therapeutic strategies [93, 94]. It is also possible to use AI to identify these different subtypes. This is certainly a very promising topic, but real-world studies and multicentre collaborative clinical research are needed for efficacy validation of subtyping-based precision treatments.

A. De Michele discussed the use of CDK4/6i. Targeted therapy with CDK4/6i in addition to ET has been widely studied in BC. Current guidelines approve indications for adding CDK4/6i to ET in these cases: Ribociclib in N0 BC if T≥3 or T2/G3 or G2 and RS>25 or KI67≥20%, and in N+ BC [95]. In these patients, adding Ribociclib 400 mg/day (days 1–21 of every 28-day cycle) for 3 years to adjuvant ET improves iDFS. Abemaciclib shows benefit in iDFS and DRFS in patients with ≥4 positive LN or 1–3 positive LN only if T≥3 or Grade 3 [96]. The benefit of adding Abemaciclib to ET appears to exist regardless of menopausal status and first ET, with a numerically greater benefit in the premenopausal compared to the postmenopausal population [80]. The ultimate goal is to optimise and personalise therapy. Ongoing trials will help in this, for example, the ADAPTcycle trial to evaluate ET plus ribociclib versus CT in intermediate-risk ER+/HER2- early BC.

M. Kok focussed his presentation on immunotherapy. Current ASCO/CAP recommendations define ER+ tumours as having ≥1% ER expression and tumours with ER expression between 1% and 10% as ER low-positive tumours. Early-stage BC with low-positive and intermediate-positive (10%–50%) ER expression have more similarities in immune biology to TNBC than to their highly ER+ counterparts, based on tumour-infiltrating lymphocytes (TILs) and PD-L1 expression. From a therapeutic poit of view, there are studies that seem to demonstrate that, also from a therapeutic perspective, ER low-positive BC could benefit from treatments similar to TNBC, for example, with the addition of pembrolizumab to NACT followed by adjuvant pembrolizumab [97] or the addition of nivolumab to NACT [98]. The gene expression profile could also play an important role in this type of patient, as seen, for example, in the case of patients with MammaPrint ‘ultra-high’ (MP2) who showed improved rates of pCR with durvalumab/olaparib added to standard NACT [99].

## Session VI: Bringing artificial intelligence to the BC clinic

We are only at the beginning, but in the near future, AI will likely become increasingly important in clinical practice, as discussed by J. Kather. The progression of individualised cancer care necessitates the development of a larger number of more accurate biomarkers for predicting treatment response, the identification of new targets in cancer cells and an accelerated drug development platform to facilitate matching targets with drugs and predict the risk of recurrences. Here, AI could be extremely useful. To fully realise the potential of AI in furthering precision oncology, several key challenges need to be addressed, such as the integration of diverse data types (e.g., imaging, genomics and clinical data), the development of interpretable and transparent AI models and the establishment of standardised protocols for data sharing and model validation. Moreover, closer collaboration between AI researchers and clinicians will be essential to guarantee that AI-based tools are clinically applicable and tailored to the needs of patients and healthcare systems [100, 101].

G. Fastner also expressed his views on the utility of AI. AI in fact can also assist in radiation oncology during all the various steps: the initial treatment-decision making (patient evaluation and dose prescription), the treatment planning (treatment simulation, image segmentation and dosimetric treatment planning), the treatment set-up and delivery (scheduling, image guidance, and motion management and adaptive treatment) and the completion of treatment (response assessment and follow-up care, toxicity prediction and management) [102]. But what is the clinical relevance of all this? For example, to compensate for the scarcity of infrastructure, to facilitate a time-consuming and technically complex cancer treatment, to decrease the lack of cancer care with RT, especially in low- and middle-income countries and to positively affect survival endpoints. These aspects are very promising, but at the moment, there are limitations that require implementations for use in clinical routine, such as the fact that software must be evaluated by medical authorities, technical development requires costs, and there is limited access to high-quality datasets.

## Session VII: Is it time to update our imaging and diagnostic approach in early BC

I. Rubio clarified the use of PET-CT in staging. Although there are differences depending on the various guidelines (e.g., NCCN, ESMO, ABC and EANM/SNMMI), in stage I, where the risk of distant metastases is very low, PET imaging, in addition to having limited utility, may lead to false positive findings [103]. In stage IIA, PET-CT may be useful, but there is not enough strong data to recommend routine use in this group of patients. Specifically, PET-CT can be recommended for baseline staging in IIB, preferably before surgery, and stage III, including inflammatory BC [104]. In the neoadjuvant setting, the role of PET is primarily valuable for axillary evaluation, but it is not very sensitive at the end of treatment to reveal residual primary tumour tissue [105]. The potential effectiveness of PET-CT to predict early pCR and patient outcomes, especially for TNBC and HER2+ BC, is interesting [41]. Furthermore, it is worth considering, in addition to the clinical stage, the tumour subtype. For example, FDG uptake differs significantly, with higher FDG uptake in TNBC than in ER+ BC [106], and the histological type, as the impact of PET-CT on systemic staging may be lower in ILC. In these cases, potentially FES-PET and Gallium-68 Fibroblast Activation Protein Inhibitor Positron emission tomography may be a better option.

And what about breast MRI? B. Gulluoglu focused his presentation on the role of MRI in the upfront surgery setting. Despite many potential benefits in using MRI, such as increased diagnostic accuracy of local staging, planning of more accurate surgery and decreased re-operations, there are potential disadvantages, such as a higher number of unnecessary mastectomies and increased unnecessary visits for further imaging and biopsies, which can lead to false positives and thus delay the timing of surgery. In current guidelines, MRI in upfront surgery is recommended, particularly in invasive ILC and to identify potential additional lesions in patients with dense breasts. In reality, critically reviewing the literature, it appears that many studies often have an inadequate representation of young patients with dense breasts and ILC cases (e.g., COMICE, MONET, Finnish, POMB and BREAST-MRI). All these studies found similar reoperation rates in both arms (MRI and no-MRI), except for the POMB study. The final mastectomy rates were similar in all studies except in the BREAST-MRI study. MRI indicated contralateral breast surgery in 2%–3% of patients. Furthermore, there are similar local recurrence (LR), local-regional recurrence, local recurrence-free survival and contralateral recurrence rates in the MRI and no-MRI arms. There is also no convincing scientific evidence either supporting or opposing the use of MRI in patients with ILC [107] and not even in younger patients with or without dense breasts [108, 109]. Based on these considerations, the use of preoperative MRI should probably be reconsidered, and it appears prudent to perform it only in well-selected patients.

J. Huober began her presentation with the question, ‘Is it time to re-think our approach in BC follow-up?’. Current goals of surveillance in BC are represented by the early detection of potentially curable events like LRs, contralateral cancer and screening for secondary tumours. In clinical practice, probably not yet. In fact, just as we do not treat our patients with drugs until efficacy has been proven in clinical trials, the same principles should be applied to survivorship and radiographic surveillance. However, we should probably focus on this question to update the surveillance guidelines, which, at present, are still based on randomised trials conducted 30 years ago [110, 111], considering, for example, that in these years, the treatment of early BC has improved significantly, diagnostic imaging has notably improved, local and systemic therapies have improved and new techniques related to molecular medicine have emerged. We could therefore integrate imaging with new techniques that allow ultra-early detection of relapse in asymptomatic patients, such as ctDNA screening [16].

## Session VIII: Hereditary BC

S. Paluch-Shimon discussed genetic tests. Approximately 5%–10% of BC are associated with a germline pathogenic variant (PV). The risk of developing BC in these patients depends on both the gene involved, the type of genetic mutation and age. The most common PVs are represented by BRCA1/2 mutation carriers, but there are PVs in other genes, particularly PALB2, ATM and CHEK2, that are associated with an increased risk of BC sufficient to justify altered medical management according to guidelines, such as increasing breast screening. Regarding the PV-positive rate, data suggest that it is higher among women who were diagnosed before 40 years, it does not change appreciably between the ages of 40 and 59 years, and it decreases among women who were diagnosed after 60 years, decreasing with increasing age [112]. The majority of international guidelines still focus on a risk-adapted approach based, for example, on age, family history, BC biology and ancestry. However, there is evidence to support multigene testing for BC susceptibility genes BRCA1/BRCA2/PALB2 in all BC patients, as this strategy can substantially reduce future malignancies [113]. The clinical utility of performing the genetic test is represented by the possibility of optimising treatment, such as the addition of adjuvant Olaparib in BRCA mutation carriers [114], and also the risk management of future malignancies, like contralateral BC, tubo-ovarian cancers and pancreatic cancer [115]. In the near future, concerns about genetic services, interpretation of variants of uncertain significance and appropriate risk assessment will likely be resolved by AI.

K. Metcalfe focused on risk-reducing mastectomy (RRM). BC risk surgery, including contralateral BC, needs to be considered when making surgical prevention decisions. In the case of BRCA1/2 mutation carriers, performing bilateral RRM reduces BC incidence and BC mortality, but further follow-up is needed to estimate the mortality reduction with greater precision [116]. On the other hand, risk-reducing contralateral mastectomy in patients affected by BC reduces contralateral BC incidence, but its effect on BC mortality is unclear [117, 118]. Important and more definitive future evaluations will derive from longer follow-up, with consideration of other genes such as ATM and CHEK2 [119].

J. Boughey discussed risk-reducing surgery through the so-called ‘conservative mastectomies’: skin-sparing mastectomy and nipple-sparing mastectomy (NSM), which are oncologically safe in appropriately selected patients. However, rates of BC development after NSM for risk reduction (bilateral prophylactic mastectomy or contralateral prophylactic mastectomy) in both BRCA-mutation and non-BRCA mutation carriers are limited, and longer follow-up is needed [120]. It is important that the surgery is performed by experienced surgeons with adequate thin flaps. After mastectomy, breast and nipple sensation are significantly diminished, with a significant negative impact on patients' QoL [121]. The implementation of new techniques such as Nerve-sparing or Nerve Grafting Mastectomy [122] or robotic mastectomy could lead to a reduction in this complication, but further data is needed.

Are there alternatives to RRM? C. Singer discussed this topic. Current guidelines suggest increasing breast screening [123]. In this context, in patients with BRCA1 mutations, for example, intensified screening with MRI reduces BC mortality. Further studies of patients with BRCA2 mutation carriers are needed to ascertain if these women obtain the same benefits [124]. The use of TAM or raloxifene may be an effective risk-reduction option for BRCA mutation carriers, but further studies with longer follow-up are necessary [125]. There is an international ongoing trial investigating the preventative effect of denosumab in healthy BRCA1 germline mutation carriers (BRCA-P trial). Risk-reducing salpingo-oophorectomy (as well as RRM) in young BC patients is associated with improved DFS, BCFI and OS, but it is necessary for healthcare providers to weigh the benefits and risks of these procedures, as they can lead to infertility and early menopause [126]. Lifestyle factors, such as physical activity and non-smoking, are important in reducing the risk of BC in BRCA mutation carriers [127, 128].

## Session IX: Optimising locoregional management: the axilla

Can we avoid upfront axillary surgery? Urban C. attempted to clarify this. At present, sentinel lymph node biopsy (SLNB) is still widely used, but it is probably time to incorporate into clinical practice what important studies have shown. Thus, in the case of N0 on preoperative US, which has a negative predictive value of 95.1% to infer low tumour burden [129], we can apply the SOUND and INSEMA studies. In cases of suspected or limited axillary involvement during preoperative US, as well as in cases of significant axillary involvement but negative fine-needle aspiration or core biopsy, we can perform SLNB and, in case of positivity, apply various studies, e.g., ACOSOG Z0011, SENOMAC, AMAROS and SINODAR. The omission of axillary surgery should not, however, mean an increase in other therapeutic modalities, such as RT; therefore, this de-escalation requires an effort in multidisciplinary shared decision-making [130]. More data are needed for SLNB omission in: ILC, G3, T2, premenopausal patients, mastectomies, and in patients with TNBC and HER2+ BC candidates for upfront surgery. For the near future, we expect more decisions based on genomics and AI.

And what about SLNB in particular situations? M.J. Cardoso discussed this. Specifically, in inflammatory BC, where the lymphatic drainage differs from other BC types, there is currently no prospective evidence to support de-escalating axillary treatment in either N0 at presentation or radiological N0 after NACT cases. Therefore, ALND (plus mastectomy without immediate reconstruction) remains the standard approach [131]. In the case of LR, it is necessary to consider that staging with PET-CT can be helpful, but there is not enough evidence to replace surgical staging. Data seem to highlight that, in patients with ipsilateral BC recurrence, receiving surgical axillary staging was associated with better survival [132]. If SLNB is feasible, previous mastectomy and ALND are not a contraindication, but the identification rate is lower [133]. If the SLNB fails, in case of negative staging, the standard of care for now is ALND. In de novo stage IV BC, the role of axillary surgery has not been prospectively evaluated independently of primary tumour surgery, but it appears unlikely to provide a survival benefit [134]. In the context of oligometastatic disease, where a curative approach is considered, the management of the axilla should follow the same principles as in early-stage BC.

W. Weber, in his presentation, emphasised axillary surgery after NACT. In cN-, perform SLNB with a single tracer [135] and omit ALND when ≥1 Sentinel lymph node (SLN) is negative [136]. In cN+ with a good response to NACT, imaging cannot replace the SLNB procedure [137], but when nodal-pCR is determined by SLNB or targeted axillary dissection (TAD), ALND can be safely omitted [138, 139]. In this setting, both TAD and SLNB are safe [140]. In cN+ and residual disease after NACT, there are important ongoing trials (A011202 ALLIANCE, TAXIS), but results are still 4–5 years away. In the meantime, while prospective trial results are awaited, some data [141] suggest that ALND may not be necessary for all patients with residual nodal disease after NACT. Some studies show that, in selected patients with a low volume of nodal disease after NACT, SLNB + image-tailored axillary surgery and adjuvant RT may be sufficient for local control of the axilla [142, 143]. Results from the retrospective ICARO study, after 5 years of follow-up, do not support routine ALND in all patients with ypN0(i+); in this study, the mean number of SLN with ITCs was 1.2 [144]. The microNAC trial seems to support further de-escalation of surgery in residual micrometastases in selected patients; results will be available soon. Another retrospective study, MACRONAC, is planned to evaluate the association between the omission of ALND and recurrence by volume of nodal disease and biological subtype in patients with residual nodal macrometastases.

A. Munshi spoke on nodal irradiation after NACT. Currently, regional nodal irradiation is decided based on clinical stage, tumour response and the extent of axillary surgery. Results from randomised trials will guide future decisions, such as NSABP-051 for patients converted to N0 post primary systemic treatment; ADARNAT, TAXIS, for patients who remain N+ after primary systemic treatment; and OPBC-10/NOAX for patients N+ undergoing upfront surgery. A balance between axillary surgery and axillary irradiation is necessary; a multidisciplinary approach is fundamental [145]. An important aspect to consider for personalised treatments is fractionation. While 15 fractions have become the current standard, based on scientific data such as those derived from START and other related trials [146], the next step will be to understand if it is possible to extend the indication to further hypofractionation, as evidenced by FAST-FORWARD [147].

## Session X: Designing clinical trials that are patients-centered, purposeful and pragmatic

M. Regan discussed the strengths and limitations of clinical trial design.

Good clinical trials are critical to the practice of evidence-based medicine, and the first step is asking well-built clinical questions [148]. The clinical trial design process is challenging, but there are tools such as SPIRIT, which provides evidence-based recommendations for the minimum content of a clinical trial protocol and outlines recommendations in a 33-item checklist and figure; and PRECIS-2, which helps trialists designing clinical trials consider where they would like their trial to be on the on the pragmatic (estimates intervention effect in usual clinical practice)/ explanatory (shows intervention works under ideal conditions) continuum.

D. Cameron reiterated the importance of the fact that clinical trials must answer a question, test a hypothesis, and therefore, patient enrolment must try to reflect this hypothesis. Furthermore, enrolment should also be as aligned as possible with the ‘real clinic population’ and not only with an ‘ideal population.’ Rarely, however, is it possible to ‘personalise’ the results by taking into consideration, for example, comorbidities or the onset of toxicities that determine an alteration of the patient's lifestyle and that would require a reduction or delay of treatment. Certainly, a shared-decision patient/physician approach is important, with emphasis, for example, on the relationship between efficacy and toxicity of what we are testing, as each patient is different from another.

F. Pignatti discussed the usefulness of stated preference studies in drug regulation, which, along with other methods such as focus groups and expert opinions, have the potential to become an important tool for gathering patient views in a systematic way to inform regulatory and treatment decisions. Indeed, for example, there is data showing that attitudes towards PFS are spread out and associated with individual differences in the willingness to trade between toxicity and PFS, hence, the importance, once again, of patient communication [149]. Furthermore, research focusing on optimising the use of health technologies in clinical practice, known as 'treatment optimisation research,' should be implemented. This is because when new treatments enter the market, there are often many uncertainties for doctors and patients, such as how to employ these new treatments in clinical practice by integrating them with those already available, optimal dose and duration, and which patient population can derive the maximum benefit [150].

T. Spanic clarified how it is important to consider the patient's perspective within clinical trials. To do this, it would be useful, for example, to involve patients early in trial design, address patient-relevant questions, recognise differences between patient and physician perspectives (e.g., side effects and their impact on patients' daily lives), minimise the burden of hospital visits, incorporate meaningful QoL questionnaires and ensure treatment protocols align with real-world patient needs and preferences.

## Session XI: Addressing the needs of young BC patients

C. Saura presented the topic of BC in pregnant patients. Pregnancy-associated cancer encompasses two different entities: BC diagnosed during pregnancy, with a prognosis comparable to non-pregnant patients according to age and stage; and BC diagnosed in the postpartum period, with a worse prognosis [151]. When possible, treatments for pregnant women with BC should be non-inferior to standard treatment, but dedicated considerations are necessary. For example, regarding imaging, we can perform: US, mammography and chest X-ray with abdominal shielding, and MRI without gadolinium. CT scans, bone scintigraphy and PET should be avoided; tumour markers are not informative during pregnancy [152]. Regarding surgery: BCS and mastectomy have essentially similar indications as in non-pregnant women. Reconstruction, preferably with an expander, can be safely performed. For SLN, the use of technetium with local injection 2 hours before surgery is recommended to minimise radiation exposure. Blue dye, however, should be avoided due to potential allergic reactions. RT in general is recommended to be postponed to the postpartum period; this should be considered in planning the surgical choice. CT is contraindicated in the first trimester due to the high risk of miscarriage and congenital malformation. In the second and third trimesters, standard anthracycline-taxane-based regimens can be administered as in the non-pregnancy setting. Carboplatin can be given safely. Immunotherapy for TNBC or anti-HER2 therapy for HER2+ BC should be deferred until after delivery. Supportive treatments such as ondansetron, metoclopramide and G-CSF can be used. Consider that after 35 weeks of gestation, weekly schedules are advisable and can be continued until closer to delivery. The use of genomic tests could be interesting to help identify low-risk BC, but studies are lacking in this setting. ET is contraindicated during pregnancy because it may induce abnormalities in the development and function of the reproductive tracts and congenital malformations [151, 152, 153]. Whenever possible, it is preferable to target full-term delivery (starting from the 37th week). Breastfeeding is contraindicated only if the patient has to continue systemic therapy or RT [154].

Fertility preservation and pregnancy after BC were the topics of discussion by M. Lambertini. All cancer patients of reproductive age should receive oncofertility counselling as early as possible in the treatment planning process, as there are several options to offer patients, such as embryo/oocyte cryopreservation, ovarian tissue cryopreservation and GnRHa during CT to reduce the rate of premature ovarian insufficiency [155, 156, 157]. Recent data have provided reassurance on the safety and feasibility of pregnancy in BC survivors, regardless of the hormone receptor status of the disease and even in BRCA carriers. It is necessary to consider that oncological treatments can be teratogenic, and therefore, contraception during treatments and an adequate period of washout are mandatory. Actual guidelines suggest waiting to try to conceive: at least 1 year after completion of CT, 7 months with trastuzumab, 3 months with TAM (although in some countries, this interval has been extended to 9 months); there is limited data available on immune checkpoint inhibitors (ICIs) and new agents [158, 159]. The POSITIVE trial evaluated the safety of suspending ET in patients after having completed more than 18 but less than 30 months of treatment. After 3 months of washout, women are allowed to conceive; it seems useful to consider a total-body restaging procedure before attempting pregnancy. The interruption was planned for a maximum of 24 months. In all these patients, resumption of ET after delivery is recommended [160, 161]. Breastfeeding appears to be oncologically safe after BC, even in BRCA mutation carriers [162, 163].

L. Michel discussed sexual health in BC survivors. What we must consider is that sexual health is an essential and often neglected aspect in BC patients. The prevalence of sexual concerns (poor body image, poor sexual functioning, poor sexual enjoyment, sexual inactivity) seems frequent, persistent and insufficiently addressed [164]. The main mechanisms causing sexual dysfunction in BC patients include emotional distress (such as anxiety and depression, potentially leading to restriction of sexual desire and self-esteem), body image alterations (surgery/RT, alopecia, loss of nipple/breast sensation and treatment-related weight gain) and premature menopause [165]. ET may lead to dysfunction, including vaginal dryness, reduced libido and dyspareunia [166]. There are various therapeutic strategies for genitourinary menopause symptoms, such as water/silicone-based vaginal products, vaginal laser, vaginal dilators, and, if these are ineffective, local estrogen therapy appears to be safe. However, it is recommended to use the lowest effective dosage and an appropriate individual risk-benefit assessment [167–172].

## Session XII: Systemic therapy for early TNBC

S. Loi focused on the correct use of immunotherapy. Early-stage TNBC contains large amounts of T cell infiltrate. The use of PD-1/PD-L1 inhibitors with CT in both neoadjuvant and adjuvant settings, in some cases, improves pCR rate, EFS and OS [173]. Current evidence supports the use of Pembrolizumab in both settings, with ongoing studies examining de-escalation by not using adjuvant pembrolizumab for patients with pCR [174]. In the pure adjuvant setting, the addition of the immune therapy drug atezolizumab to CT after surgery did not provide benefit. This suggests that a lower micrometastatic tumour burden or the absence of residual cancer is not enough to trigger a T cell response [175]. The optimal duration of PD-1 inhibitors, both pre- and post-surgery, in early TNBC is an evolving field. In the neoadjuvant setting, the duration is probably less important than achieving pCR. In the post-surgery setting, for patients with pCR, there is no rationale for continuing immunotherapy. A longer duration of immunotherapy is associated with more immune-mediated adverse events, particularly endocrinopathies. The onset of immune-related adverse events can occur even long after (up to 41 months) the start of treatment with PD-1/PD-L1 inhibitors. Immune biomarkers can already help tailor shorter duration NACT + immunotherapy (Neo PACT, NeoN) that can achieve high pCR rates, and thus, de-escalation prospective studies should incorporate biomarkers (e.g., TILs and ctDNA). In the near future, strategies will probably be found to identify and mitigate immunotherapy-related toxicity, for example, using future PD-1 agents that target tumour cells and not normal cells.

W.F. Symmans discussed the important role of RCB. It would be necessary to standardise the routine quantification and reporting of RCB after NACT, as there is a long-linear relationship between RCB score and prognosis [45]. The prognostic surrogacy of RCB was irrespective of neoadjuvant treatment and provides an assessment of residual risk that appears to be clinically meaningful and could inform a patient’s subsequent adjuvant treatment. Furthermore, investigational treatments that shifted the distribution of RCB values in I-SPY2 suggested subtype-specific differences in patterns of RCB shift that are hypothesis-generating, and they also had longer EFS in an exploratory analysis. Thus, the survival benefit of a specific treatment may be reflected in changes to the RCB distribution, with a larger shift implying a greater probability of efficacy [176]. TILs RD (residual disease) levels in TNBC treated with NACT are significantly associated with improved RFS and OS and add further prognostic information to RCB class, particularly in patients with moderate RCB [177].

L. Carey attempted to answer three important questions. The first one: Is it possible to administer the ICI only in the adjuvant setting? Considering the results of the KEYNOTE-522 trial with a higher percentage of pCR among patients who received Pembrolizumab + CT in the neoadjuvant setting, the answer is probably ‘no’. The answer continues to be ‘no’ even when analysing two other trials: the A-BRAVE trial shows that the addition of ICI (Avelumab) for 1 year does not significantly increase DFS [178], similarly, the ALEXANDRA trial does not show an improvement in DFS with the addition of Atezolizumab in the adjuvant setting [175], for the mechanism already highlighted by Prof. Loi during her presentation. Turning to the second question: Is adjuvant therapy necessary in patients who achieved pCR? In GeparNuevo, the addition of Durvalumab to NACT, despite a modest pCR increase, significantly improved OS; in this case, ICI was not continued after surgery [179]. There are ongoing trials such as OPT-PEMBRO and OptimICE, whose primary endpoint is the evaluation of EFS in patients who achieved pCR with Pembrolizumab + CT in the neoadjuvant setting, without continuing pembrolizumab after surgery. Pending these data, the standard is the completion of adjuvant Pembrolizumab, if patients are tolerating it. Finally, the last question: are more drugs needed in residual disease? In these high-risk patients, probably yes. For example, in the CREATE-X trial, after standard NACT containing anthracycline, taxane or both, the addition of adjuvant capecitabine therapy at a dose of 1,250 mg per square meter of body-surface area, twice daily conventional schedule for 6 months, was safe and effective in prolonging DFS and OS among patients with HER2-negative BC who had RD [180], but other clinical trials such as GEICAM/2003 failed to show a statistically significant improvement in DFS by adding capecitabine in the adjuvant setting [181]. Among patients with high-risk, HER2-negative early BC and germline BRCA1 or BRCA2 PVs, adjuvant Olaparib was associated with significantly longer DFS [114]. In essence, for now, it is unknown whether adjuvant capecitabine adds benefit in patients receiving adjuvant continuation of their ICI or olaparib; no efficacy results are available for either of these combinations in the adjuvant setting [106]. There are several ongoing trials, such as OptimICE-RD (AFT-65)/ASCENT-05, SASCIA and TROPION-Breast03, that may help to clarify.

Do all TNBCs require CT? H. Rugo discussed this. CT is recommended for T1c/N0 or greater TNBC. Data in pT1b pN0 are unclear, but probably for most of them, adjuvant CT is suggested. The majority of patients with pT1a/N0 TNBC do not benefit from adjuvant CT, but probably, it is advised in highly selected patients, such as young patients with a high grade [182]. Patients with low-grade, good prognosis, rare TNBC subtypes (e.g., adenoid cystic, secretory carcinoma, apocrine, low-grade fibromatosis-like carcinoma and low-grade adenosquamous) do not need CT [106, 183]. At present, we are probably still over-treating at least some TNBC. Help to better stratify treatments could come from the use of biomarkers such as TILs levels, which have a prognostic value [184, 185]. There are ongoing trials, like, for example, NeoTRACT, which investigates de-escalating NACT based on the degree of pre-treatment TILs enrichment. In fact, this is positively associated with pCR in TNBC and may be useful to identify patients for whom de-escalation of NACT might be acceptable.

A. Tutt focused on PARP inhibitors (PARPi) and new approaches to homologous recombination deficiency (HRD). PARPi has been demonstrated to exhibit anti-tumour activity in individuals whose cancers have a defect in the HR DNA repair pathway. HRD populations may be larger than populations with BRCA1/2 mutations. For example, 60% of TNBC have HRD and could potentially benefit from PARPi [186]. The OlympiA trial showed that 1 year of adjuvant Olaparib in patients with germline mutations improves iDFS and OS [187], but resistance to PARPi can develop. For example, BRCA1/2 reversion mutations are observed in ctDNA in 60% of patients who develop resistance and were the most prevalent form of resistance. The cause of the reversion appears to be driven by a polymerase theta-mediated process, and this nature of the resistance mechanism may have implications for the use of DNA polymerase theta inhibitors and other agents that may be synthetic lethal with 53BP1/Shieldin complex mutations or non-HR-related aspects of BRCA1/2 deficiency [188]. There are ongoing trials testing polymerase theta inhibitors (e.g., MOMA 313, GSK4524101 and ART6043). Another potential target for new drugs is represented by USP1 inhibitors. USP1 is required for fork protection in BRCA1-deficient cells. There are trials testing these inhibitors (e.g., KSQ-4279). Finally, other potential drugs could be represented by ATR inhibitors (tuvusertib) and WEE1 inhibitors (azenosertib).

S. Dawood emphasised the side effects associated with the use of ICI. ICI is an increasingly important in BC treatment, and as more patients are treated with ICI and live longer, we will see an increasing incidence of both acute and chronic adverse events, which we need to manage. Most of the toxic effects are reversible, aside from effects on the endocrine system, which may be permanent [189]. Furthermore, ICI can impact immediate and future fertility, and therefore, fertility preservation should be strongly advised [190]. The precise pathophysiology underlying immune-related adverse events is unknown but is believed to be related to the role that immune checkpoints play in maintaining immunologic homeostasis [191]. More studies are needed to better elucidate the mechanisms underlying the development of these adverse factors, in order to identify and manage them multidisciplinary by developing personalised treatment strategies.

## Conclusion

The final day focused on the consensus panel, the true highlight of the St. Gallen congress.

The purpose of this consensus, chaired by leading international experts, is to develop guidance for the majority of common presentations and clinical scenarios in early-stage BC. The various aspects of the diagnosis and treatment of BC were discussed. A summary of these discussions is presented below.

The conclusions are the most important and most-read part. To make it easier to read, I think it would be better to make the different sections more prominent. I leave the method up to you, but I'm underlining the sections in green. Whenever possible, in patients without high risk and without contraindications to RT, BCS is preferred instead of mastectomy. However, patient preference is also important in shared decision-making.

SLNB can be omitted in upfront surgery for postmenopausal patients with T1, N0, luminal BC. Currently, there is insufficient data to omit it in patients with TNBC and HER2+ BC, and also in ILC, even when preoperative axillary US is negative.

Following NACT, in the presence of MRD in the axilla, ITC or micrometastasis in favourable biology, the panel predominantly advises against ALND, instead recommending nodal irradiation. However, in the specific case of ITC in luminal BC, RT may be omitted.

We are less comfortable with a finding of 1/1 or 2/2 positive SLNs out of the total sampled, but in the case of 1 macrometastasis in 4 SLNs after NACT with a good clinical response, 58% of the panel expressed a preference for nodal irradiation over ALND especially in luminal BC or HER2+. For TNBC with residual macrometastases and in inflammatory BC ALND remains the gold standard.

The standard treatment schedule of RT should now be considered moderate hypofractionation with 15–16 fractions. Potential de-escalation with ultra-hypofractionation can be considered in post-menopausal patients with low-risk BC (FAST-FORWARD) and even a viable single-modality treatment, RT or ET (LUMINA, IDEA, EUROPA).

Post-mastectomy radiation therapy is recommended for high-risk BC, with irradiation of the chest wall and regional LN, if it is involved. According to the panel, RT is preferable to ALND for 1 or 2 macrometastatic SLNs in upfront surgery.

Hereditary BC: Genetic testing is an important tool, but it must be offered ‘judiciously’ and not routinely to all patients, for example, in women with newly diagnosed a high aggressive BC or in patients aged 50 or less, male BC or strong family history. Regarding risk-reduction surgery, the panel agrees for patients with PVs in BRCA1-2, PALB2 upto 50–55 years old. For older women or those with PVs of ATM, CHECK2, BARD1 or RAD51 genes, screening is preferred. NSM is the surgical procedure of choice, preferably performed by surgeons with high expertise.

Survivorship: Considerable emphasis was placed on patients' QoL. For example, to prevent neuropathy in women receiving taxane-based neo/adjuvant treatment, the majority of the panel recommends routinely offering cooling gloves. Compression gloves are also useful, but appear to be less effective. Alternatively, they can be used in combination.

DCIS: At present, the recommended treatment for G2 DCIS is surgery. In case of BCS, adjuvant therapies depend on age, DCIS size and HR status: in healthy non-elderly patients with DCIS ≥10 mm HR+, it is advisable to combine RT and ET. In women over 70, a de-escalation can be considered. Genomic testing for DCIS management is not routinely recommended at this time. Instead, it is preferred to base decisions on the presence/absence of necrosis, microcalcifications and grade.

ER+/HER2-BC: More than 60% of the panel recommends adjuvant CT if the likely benefit on distant recurrence-free survival is ≥5%. All panellists agree on requesting genomic risk assays for N0 tumours ≥10 mm, with 30% even recommending it for smaller sizes. For T2, N0 BC with a high RS (26–31), more than 70% of the panel favours the use of CT, particularly anthracycline-free regimens (docetaxel-cyclophosphamide) or even lower in the case of N1; taxane-anthracycline for RS ≥ 31. However, it is necessary to use clinical judgment to individualise therapy. For example, in premenopausal patients, consider CT in N0 even with RS lower than 26 and OFS if RS ≥ 16. In N+, 67% of the panel recommends CT in addition to ET for RS ≥16. Standard ET duration is 5 years, however, in high-risk BC, extending ET beyond 5 years should be considered: a treatment duration of 7 to 8 years appears sufficient for the majority of these patients, 10 years for ≥ 3 positive lymph nodes. Targeted therapy with CDK4/6i in addition to ET should be considered in higher aggressive BC.

HER2+ BC: In N0 disease, over 90% of the panel recommended NACT plus anti-HER2 therapy for tumours ≥ 20 mm, with 40% advocating this approach even for tumours ≥ 15 mm. In stages II and III disease, 75% of the panel agreed on an anthracycline-free regimen combined with dual blockade TCb/HP (Docetaxel, Carboplatin, Trastuzumab, Pertuzumab), with 60% supporting this even in cases of inflammatory BC. Patients with no-pCR after NACT plus anti-HER2 therapy should receive adjuvant treatment with T-DM1, according to over 70% of the panel.

For patients with T1b, N0 disease, upfront surgery followed by adjuvant 12 weeks of paclitaxel plus 1 year of trastuzumab was deemed the correct therapeutic choice by 90% of the panel, rising to unanimous agreement in cases of T1c, N0 disease. Pertuzumab addition is considered only in N+. In cases of only foci of microinvasive cancer, over half of the panel did not recommend paclitaxel/trastuzumab. For risk stratification, various tools are available, such as omics integration or HER2DX, which 50% of the panel found useful. However, these tools are not yet widely utilised in clinical practice.

TNBC: NACT is the standard of care for T1c-4/N0 or N+. Concerning immunotherapy, the addition of pembrolizumab, according to 70% of the panellists, is indicated when the tumour size is >20 mm and, pending the results of ongoing trials, is currently continued after surgery even in cases of pCR (approximately 90% of the panel). In the case of durvalumab, it is not continued in the adjuvant setting (GeparNuevo). Immunotherapy should not be used in the adjuvant setting alone. If patients, with stage 2 or 3, have contraindications to the use of checkpoint inhibitors, nearly 90% of the panel recommends TCb/AC (Docetaxel, Carboplatin, Anthracycline, Cyclophosphamide) administration. Patients with residual disease following NACT should receive adjuvant capecitabine for 6–8 cycles. Furthermore, 75% of the panel agrees to continue immunotherapy and add capacitabine. In BRCA1/2 BC immunotherapy and concurrent olaparib.

The panel, with 82% agreement, recommends treating ER-low tumours equivalently to TNBC, with the adjunct of pembrolizumab to NACT.

In N0, 100% of the panel recommends adjuvant CT for tumours >10 mm; 76% even if the size is >0.5 mm, excluding adjuvant CT only in cases of pT1a.

Oligometastatic BC: For limited metastatic disease with highly effective treatment options and/or a favourable initial response to therapy, nearly 90% of the panel agreed on considering definitive local-regional treatment. For patients presenting with de novo oligometastatic disease and a clinically negative axilla, 70% of the panel recommended performing SLNB.

The 19 St. Gallen Congress addressed the current standard of care while exploring emerging aspects of early BC treatment, highlighting the increasing importance of integrating genomic assays and AI in the near future. Our role, as physicians and researchers, must be to advance along this path and, as Dr. Giuliano emphasised at the outset, to embrace these new tools and not resist change, because ‘the fear of change is often worse than the change itself’.

## Conflicts of interest

None.

## Funding

None.
